# Construction of a genetic linkage map and QTL mapping of fruit quality traits in guava (*Psidium guajava* L.)

**DOI:** 10.3389/fpls.2023.1123274

**Published:** 2023-06-22

**Authors:** Sukhjinder Singh Maan, Jaswinder Singh Brar, Amandeep Mittal, Manav Indra Singh Gill, Naresh Kumar Arora, Harjot Singh Sohi, Parveen Chhuneja, Guriqbal Singh Dhillon, Navdeep Singh, Sujata Thakur

**Affiliations:** ^1^ Department of Fruit Science, Punjab Agricultural University, Ludhiana, India; ^2^ School of Agricultural Biotechnology, Punjab Agricultural University, Ludhiana, India; ^3^ Krishi Vigyan Kendra, Guru Angad Dev Veterinary and Animal Sciences University, Barnala, India; ^4^ Department of Plant Breeding and Genetics, Punjab Agricultural University, Ludhiana, India

**Keywords:** BLUPs, composite interval mapping, InDels, KASP, TSS

## Abstract

Guava (*Psidium guajava* L.) is an important fruit crop of the Indian sub-continent, with potential for improvements in quality and yield. The goal of the present study was to construct a genetic linkage map in an intraspecific cross between the elite cultivar ‘Allahabad Safeda’ and the Purple Guava landrace to identify the genomic regions responsible for important fruit quality traits, viz., total soluble solids, titratable acidity, vitamin C, and sugars. This population was phenotyped in field trials (as a winter crop) for three consecutive years, and showed moderate-to-high values of heterogeneity coefficients along with higher heritability (60.0%–97.0%) and genetic-advance-over-mean values (13.23%–31.17%), suggesting minimal environmental influence on the expression of fruit-quality traits and indicating that these traits can be improved by phenotypic selection methods. Significant correlations and strong associations were also detected among fruit physico-chemical traits in segregating progeny. The constructed linkage map consisted of 195 markers distributed across 11 chromosomes, spanning a length of 1,604.47 cM (average inter-loci distance of 8.80 markers) and with 88.00% coverage of the guava genome. Fifty-eight quantitative trait loci (QTLs) were detected in three environments with best linear unbiased prediction (BLUP) values using the composite interval mapping algorithm of the BIP (biparental populations) module. The QTLs were distributed on seven different chromosomes, explaining 10.95%–17.77% of phenotypic variance, with the highest LOD score being 5.96 for qTSS.AS.pau-6.2. Thirteen QTLs detected across multiple environments with BLUPs indicate stability and utility in a future breeding program for guava. Furthermore, seven QTL clusters with stable or common individual QTLs affecting two or more different traits were located on six linkage groups (LGs), explaining the correlation among fruit-quality traits. Thus, the multiple environmental evaluations conducted here have increased our understanding of the molecular basis of phenotypic variation, providing the basis for future high-resolution fine-mapping and paving the way for marker-assisted breeding of fruit-quality traits.

## Introduction

Guava (*Psidium guajava* L.) is a commercially cultivated member of the Myrtaceae family (Grattapaglia et al., 2012). Guava is indigenous to tropical America and is now flourishing in the Indian sub-continent (Nimisha et al., 2013). India enjoys a prime location for guava production. Guava fruit is popularly known as the “apple of the tropics” and is sometimes referred to as a “poor man’s apple” due to its availability, high nutritive value, and affordable prices (Sanda et al., 2011). It is the one of the most popular fruits among consumers due to its high palatability and sweet–acidic taste (Pedapati et al., 2014). The ascorbic acid content of guava fruit is five times higher than that of citrus fruits (Hassimotto et al., 2005). Owing to its high nutraceutical value, guava fruit is the best option for providing nutritional security for people in developing countries (Kumar et al., 2020). In addition, guava is very profitable, produces large yields, and can also be grown satisfactorily even in adverse soil and climatic conditions (Pommer and Murkami, 2009).

Fruit quality is a key factor for guava consumption and consumer acceptance. Fruit quality is itself not a trait; it is a phenomenon comprising the complex interactions of many physio-chemical fruit traits such as total soluble solids, titratable acidity, vitamin C, and sugars. The priority for future guava breeding programs is therefore to develop cultivars with good fruit quality, including high levels of TSS, good sugar–acid blend, high vitamin-C content, higher lycopene content, good pectin content, and good flavor (Negi and Rajan, 2007). By contrast, there is a little merit in improving yield (Dinesh and Vasugi, 2010). For the continual genetic improvement of guava, diversity at the genomic level is required (Ritter, 2012; Singh et al., 2018). Hybridization breeding enables the exploitation and transference of alleles from different genetic backgrounds for the development of better recombinants (Sohi et al., 2022). The traditional breeding of fruit crops is expensive, time consuming, and constrained by a long juvenile phase (Longhi et al., 2013). Marker-assisted breeding (MAB), or genomics-assisted breeding (GAB), offers an alternative approach to the traditional breeding of fruit crops with a long juvenile phase, allowing breeders to select fruit-quality-related traits at the seedling phase (Varshney et al., 2005; Kole et al., 2015; Baumgartner et al., 2016).

A comprehensive understanding of the genetic determinism of fruit quality is necessary to facilitate the breeding of new varieties of fruit crops (Gmitter et al., 2007; Mohsenipoor et al., 2010). Molecular marker technology combined with genetic-linkage mapping and quantitative trait locus (QTL) mining has led to the understanding of the genetic architecture and inheritance of underlying genes affecting quantitative traits (Yin et al., 2003; Hasan et al., 2021). This strategy enhances the possibilities of locating desirable alleles affecting economically important traits in fruit crops (Soto-Cerda and Cloutier, 2012). Trait mapping helps with the efficient and rapid selection of elite breeding lines and speeds up the development of novel cultivars compared with conventional breeding by uncovering masked interesting alleles from related and unrelated species to facilitate targeted introgression (Singh et al., 2014; Hasan et al., 2021). There are only a few reports of QTLs (quantitative trait loci) controlling bio-chemical traits in guava (Valdés-Infante et al., 2003; Rodríguez et al., 2007; Ritter et al., 2010; Padmakar et al., 2016). In recent years, simple sequence repeats (SSR) markers in combination with random amplified polymorphic DNA (RAPD), amplified fragment length polymorphism (AFLP), and sequence-related amplified polymorphism (SRAP) markers have been utilized for the genetic mapping of guava (Lepitre et al., 2010; Ritter et al., 2010; Padmakar et al., 2015). Due to the abundance of microsatellites throughout all the genomic components of eukaryotic organisms (Kalia et al., 2011), and features such as their multiallelic nature, automation, ease of detection, reproducibility, co-dominance, and capability for high-throughput genotyping (Liu S et al., 2013; Kumar et al., 2020), SSRs are still the chosen markers in most of the marker-assisted breeding programs in horticultural crops (Ritter, 2012; Nimisha et al., 2013).

Guava is still considered an orphan crop with reference to its genomic and /or genetic information (Mittal et al., 2020; Sohi et al., 2022). Recent advancements in next-generation sequencing (NGS) have considerably accelerated the discovery of SSRs, short insertions and deletions (InDels), and single-nucleotide polymorphism (SNP) as PCR-ready genome-wide markers through comparative transcriptomics in guava (Thakur et al., 2021). Thakur et al. (2021) identified abundant structural variations such as SSRs, SNPs, and InDels spread across the whole genome, which will be highly useful in developing functional markers for guava breeding. InDels were found to be more polymorphic than microsatellite markers (Liu B et al., 2013; Wu K et al., 2014) and received more attention because of their co-dominant inheritance, reproducibility, and easy-to-use nature (Jain et al., 2019). Kompetitive allele-specific PCR (KASP) is a fluorescence-based SNP genotyping platform efficient enough that the need for sequencing-based mapping is avoided. It utilizes the abundance of polymorphic sites to generate high-density linkage maps. (Semagn et al., 2014; Kaur et al., 2020). Thus, it has potential to increase the precision of gene tagging for the traits controlling fruit quality.

The availability of transcriptomics-based genome-wide markers lays the groundwork for the improvement of quality and agronomic traits in fruit by gene mapping in biparental populations, thus making genomic selection possible for guava. Accordingly, in this study, genome-wide InDels, KASP, and SSR markers were utilized to construct a linkage map in ‘Allahabad Safeda’ × Purple Guava and identify QTLs associated with fruit-quality traits.

## Materials and methods

### Plant material

A total of 125 F_1_ (first filial generation) plants derived from ‘Allahabad Safeda’ × Purple Guava (Singh, 2017), maintained at the college orchard of the Department of Fruit Science, Punjab Agricultural University, Ludhiana, were phenotyped for the computation of fruit–trait segregation and genotyped for the establishment of marker–trait association.

### Development of mapping population

**Figure 1:**
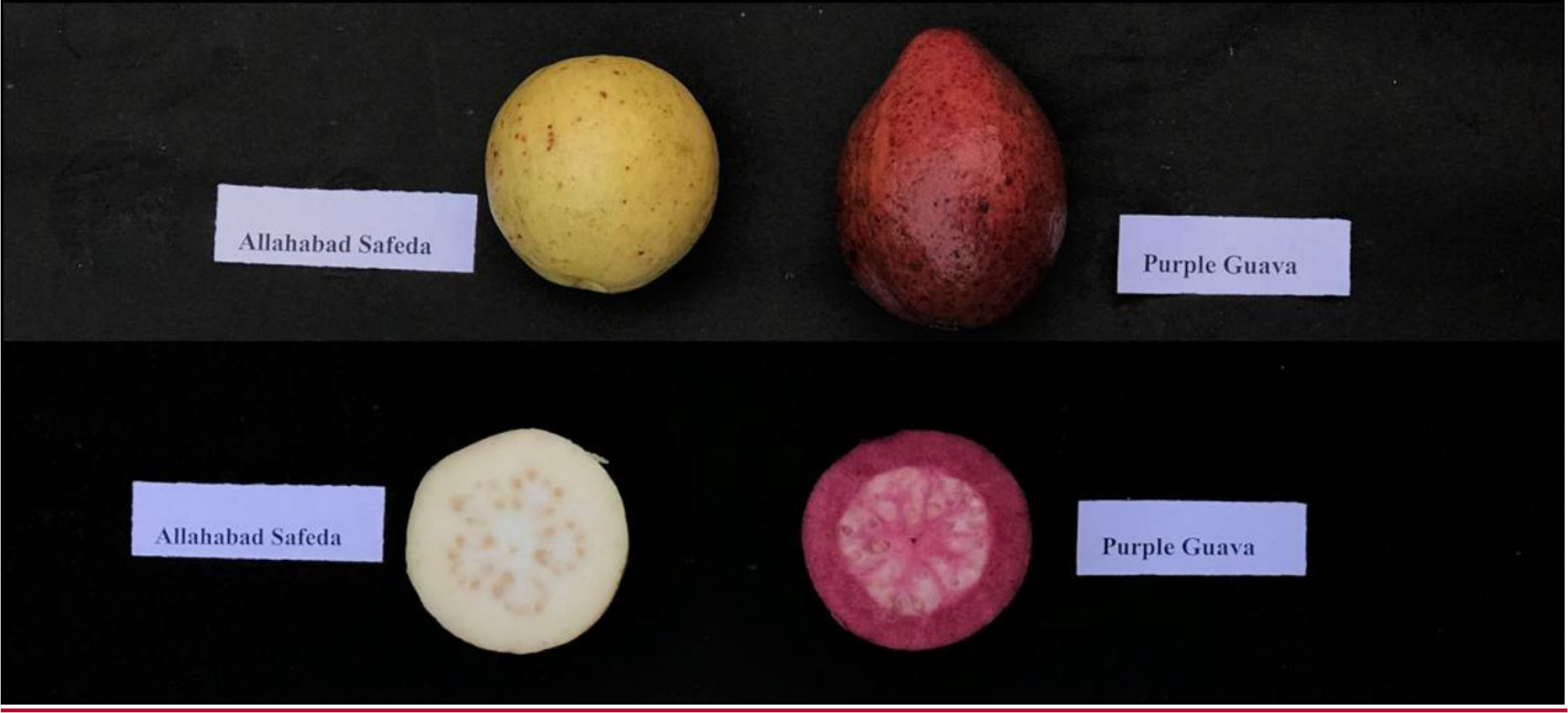
Phenotypic appearance of ‘Allahabad Safeda’ and Purple Guava.

Guava cultivars showing contrasting fruit-quality traits were selected as parents for hybridization (**Figure 1**). Six female trees aged 8–10 years were pollinated with the pollen of three male trees (aged 10 years) grown in the same orchard to develop the hybrid population (Sohi et al., 2022). The female flowers were emasculated at the balloon flower stage after removing the petals in the evening and bagging them to avoid visits by pollinators. The next morning, the emasculated female flowers were pollinated by hand between 08:00 and 10:00 using pollen from the male parent. Extracted seeds from fruits harvested at the fully-ripe stage were sown in polythene bags. Three-month-old seedlings were transplanted at a spacing of 6 m (row-to-row) × 3 m (plant-to-plant) during 2015. Trees were irrigated using the furrow irrigation method and weeding was performed at regular intervals. Fertigation dosages and other agricultural practices were followed as recommended for this region.

### Evaluation of fruit physio-chemical traits

Fruits free from visual blemishes were randomly harvested at the color-break stage from each F_1_ hybrid along with their parents for three consecutive years. Winter fruits, picked from November to January, are free from fruit fly maggots. To improve the precision of the phenotypic evaluation, fruit were sampled only from winter crops and taken immediately to the lab for biochemical analysis. Between 5 and 15 fruits were sampled (using muslin cloth) from each tagged plant for analysis of the following biochemical characteristics) in order to identify the relevant QTLs: total soluble solids (TSS), titratable acidity (TA), vitamin C (VC), total sugar content (TS), reducing sugars (RS), and non-reducing sugars (NRS). The juice of fully mature fruits was strained through muslin cloth and thoroughly stirred for the measurement of TSS is in degrees Brix (°B) with the help of a digital hand refractometer (at 20°C with correction chart). TA (%) was measured by titration of 2 mL fruit juice against a standardized N/10 Sodium Hydroxide (NaOH) solution, using phenolphthalein dye (two drops) as an indicator to obtain an end point (persistence of light pink color for at least 2 seconds), and expressed as a percentage of citric acid. Ascorbic acid content (vitamin C) was estimated from guava pulp using 2,6-dichlorophenol indophenols dye (DCPIP) visual titration assay involving a reduction reaction. Briefly, 5 g of the fruit sample was ground with about 25 mL of 4% oxalic acid. The filtrate solution was passed through Whatman No. 4 filter paper, and was collected in a 50 mL volumetric flask. The resulting solution was titrated against a standard dye until a rose pink color persisted for 5 seconds. The amount of ascorbic acid was expressed as µg/kg of fresh mass (AOAC, 2000).

### Sugars (%)

For the measurement of sugars, 5 mL of juice extract was placed in a 100 mL beaker, and 2 mL of lead acetate (45%) was added. The resulting solution was kept at room temperature for 10 minutes. After the incubation, 5 mL of potassium oxalate (22%) along with distilled water was added to make a volume of 100 mL. It was then filtered through Whatman’s filter paper No. 1. The percentage of total sugar and reducing sugars was estimated using Lane-Eynon’s titration methodology (Patel et al., 2013).

### Reducing sugar (%)

For the measurement of reducing sugar, 25 mL filtrate out of a 100 mL solution was kept overnight after adding 5 mL hydrochloric acid solution (60%), for complete hydrolysis. Samples were kept at 68°C in a water bath for 10 minutes. Thereafter, NaOH (10% and 0.1%) solution was used for neutralization, using phenol phthalein drops as an indicator of the neutralization point. Total solution used for titration was considered for calculating the percentage of reducing sugars, employing the formula


$$RS\;=\;\left(\left(0.05/V_{1}\right)\times \left(V_{2}/V_{3}\right)\times 100\right)$$

### Total sugars (%)

The remaining filtrate was added to a burette for titration using 5 mL of heated Fehling solutions (A and B) then placed in a flask and heated on a hot plate, using methylene blue dye as an indicator with a brick red color as an end point. The values noted for titration were used for calculating the percentage of total sugars with the formula


$$TS\;=\;((0.05/V_{1})\;\times \;\left(V_{2}/V_{3}\right)\;\times \;\left(V_{4}/V_{1}\right)\;\times \;100)$$

where V_1_ is the volume of filtrate used, V_2_ is the dilution made, V_3_ is the volume of juice taken, and V_4_ is the final volume made.

### Statistical analysis

The field experiment followed a completely randomized design and the analysis of variance (ANOVA) was performed using the ‘stats’ package of R (version 3.1.3, https://www.r-project.org/) in R v4.0.3 with *p ≤*0.05 and means separated using the least significant difference (LSD) test. Adjusted values on the basis of ANOVA were calculated by fitting linear mixed effects models in lme4 package v 1.1–26 (Bates et al., 2015) in R v4.0.3 (R Core Team, 2019) using


$$Y_{ik}=\mu +Year_{i}+Line_{k}+\epsilon_{ik}$$

where Y_ik_ is the trait of interest, µ is the mean effect, Year_i_ is the effect of the *i*th year, Line_k_ is the effect of the *k*th line, and Ɛ_ik_ is the error associated with the *i*th year and the *k*th line, which is assumed to be normally and independently distributed, with a mean of zero and homoscedastic variance (s^2^). For the best linear unbiased predictions (BLUPs) model, all the effects were considered as random effects. The genotypic, phenotypic, and environmental coefficient of variation was categorized according to (Deshmukh et al., 1986) and was considered as low at<10%, moderate at 10–20%, and high at ≥20%. Heritability percentage was categorized as suggested by Singh (2001) and was considered as low at< 40%, medium at 40–59%, moderately high at 60–79%, and very high at ≥ 80% (Allard, 1960). Genetic advance over mean (GAM) was categorized as high when at<20%, moderate at 10–20%, and low at< 10% (Johnson et al., 1955). The correlation of different traits was studied with simple pairwise Pearson’s correlations among traits.

### Principal component analysis and structural equation modeling

The association among different traits was identified by principal component analysis (PCA) using FactoMineR v2.4 (Lê et al., 2008) and factoextra v1.0.7 (Kassambara and Mundt, 2020) in R v4.0.3. The principal components were plotted as biplots to study the relationship among physico-chemical traits of the three environments and BLUPs for the identification of reduction in environmental effects in fitted values. Structural equation modeling (SEM) in the package lavaan v 0.6–7 (Rosseel, 2012) was calculated and visualized using the package semPlot v1.1.2 (Epskamp, 2019) to identify the direct and indirect contributors to TSS. Cluster analysis was performed using the K-means cluster analysis method with R software (Idlette-Wilson, 2018).

### Genotyping

#### Genomic DNA extraction

High-quality genomic DNA of 125 F_1_ individuals and their parents was extracted through the procedure described by Doyle and Doyle (1990) with the addition of 2% polyvinyl pyrrolidone (PVP) in cetyltrimethylammonium bromide (CTAB) buffer to account for polyphenolic compounds from the DNA samples.

### SSR/EST-InDels assay

A total of 106 SSR primer pairs, six fruit-color specific markers from the published literature (Sohi et al., 2022), and 108 primer pairs (15 genomic SSR primer pairs and 93 comparative RNA-sequencing InDel-based primers) designed from transcriptome data (Thakur et al., 2021) were used for the present study. PCR reaction mixture for EST-InDel assay comprised of 11 µL reaction with 2 µL DNA template (5ng/µL), 5 μM forward primer, 5 µM reverse primer, 0.5 µl BSA (100 mg/ml), 0.5 µl PVP (100 mg/ml), and 2 µL H_2_O (nuclease free). The reaction was performed in 384- well thermal cycler. DNA amplification was performed as follows: denaturation at 95°C for 3 min; 5 cycles of 95°C for 30 s, 55°C for 1 min, and 72°C for 1 min; 30 cycles of 95°C for 20 s, 55°C for 30 s, and 72°C for 30 s; and a final extension cycle of 72°C for 5 min and 16°C indefinitely. The amplified DNA fragments were resolved on ethidium bromide (10 mg/mL), stained with 6% non-denaturing polyacrylamide gel electrophoresis (PAGE; CBS, Scientific) for a high resolution, and visualized under a UV-transilluminator based gel documentation system after initial running at 300 V for 2 h. The molecular weight of amplicons from SSR/EST-InDels was determined based on their migration relative to a 50 bp DNA ladder. Polymorphic markers were scored as A (heterozygous in the female parent), B (heterozygous in the male parent), H (heterozygous in both parents), and X (for missing/non-amplified alleles).

### EST-SNP/KASP assay

KASP assay was performed in a reaction mixture of 4.2 µL [2.2 µL DNA (5ng/µL), 1.944 µL of 2X KASP mix, and 0.054 µL primer mix] in a 384-well format thermal cycler (Applied Biosystems) following PCR conditions: hot-start activation at 95°C for 15 min, followed by 10 touchdown cycles (95°C for 20 s then touchdown at 64°C initially and decreasing by 0.8°C per cycle from cycle 2), and then 30 additional cycles of annealing (95°C for 20 s then 57°C for 60 s). If necessary, 5 to 10 additional annealing cycles were followed for better amplification and cluster formation. Fluorescence from amplicons was visualized using a 384-well plated TECAN infinite F200 PRO plate reader at room temperature. The graphical output(s) was generated and scored using Klustercaller software (version 2.22.0.5). Based on the fluorescence signal [FAM (Fluorescein amidites) and HEX (Hexachloro-fluorescein)], homozygous alleles on the X-axis (FAM) were scored as A, homozygous alleles on the Y-axis (HEX) scored as B, whereas heterozygotes alleles (FAM/HEX) on the X–Y plot were scored as H. ROX (6-carboxyl-X-Rhodamine) was used as passive dye for signal normalization.

### Statistical analysis

#### Linkage map construction

A genetic linkage map was constructed for 125 F_1_ progenies of ‘Allahabad Safeda’ and Purple Guava with the help of QTLIci mapping software version 4.1 (Meng et al., 2015). The genotypic scores from different marker systems were used for calculating the genetic distances in cM. The allocation of markers to different linkage groups was performed *via* pairwise analysis with a specified minimum logarithm of odds (LOD) score of 3.0 and maximum recombination frequency of 0.3 (regression mapping algorithm and Kosambi’s mapping function). Markers were first partitioned into linkage groups based on the information in the published literature (Thakur et al., 2021; Sohi et al., 2022). Pearson’s chi-squared test was performed to evaluate the goodness of fit to the expected 1:1 segregation ratio for each locus (*p*-value threshold of 0.05). Markers showing distortion inconsistencies, or conflicted recombination frequencies within linkage group and/or with adjacent linkage groups, were discarded. The linkage map was graphically displayed using the MapChart program, v. 2.32 (Voorrips, 2002), according to the user’s manual. The genome coverage (GC) of linkage groups was estimated using the method of Fishman et al. (2001) and method 4 of Chakravarti et al. (1991).

### QTL analysis (establishment of marker–trait association)

QTL mapping was conducted with multiple regression analysis for composite interval mapping (CIM) using the (chromosome segment substitution lines) with chromosome segment substitution lines (CSL) functionality of QTL IciMapping version 4.1 software (Meng et al., 2015). Furthermore, the allelic effects were investigated to identify significantly associated markers with phenotypic data *via* a non-parametric Kruskal–Wallis test (with a stringent significance level of 0.005) for studying the importance of individual alleles. Stepwise regression was used to determine the percentages of phenotypic variance explained (PVE) (*R* ^2^) by individual QTL and their respective additive effects at the likelihood of odds ratio peaks. A threshold LOD score (3) was calculated using permutation tests (1,000 permutations in each case) with a 5% significance level. Negative additive effects indicated that Purple Guava alleles increased the phenotypic trait values, whereas positive additive effects meant that ‘Allahabad Safeda’ alleles increased the phenotypic trait values. QTLs detected in more than one environment were considered as stable and significant. If a chromosomal and/or overlapping interval contained more QTLs for multiple traits, then these QTLs were considered to form a QTL cluster. The overlapping confidence intervals of these QTLs were regarded as the confidence intervals of the QTL clusters. In addition, QTLs detected at phenotypic variation explained (PVE) > 10% and LOD > 3.0 were considered as major QTLs and others as minor QTLs. QTLs were assigned names beginning with an initial letter “q” followed by the trait name (in capital letters), parentage of the alleles, location of experiment, and linkage group. A number was added if two or more QTLs were identified in the same linkage group. For example, if two QTLs for TSS were detected on LG6, they were named qTSS.PG.pau-6.1 and qTSS.AS.pau-6.2.

### Differential expression analysis of genes associated with QTLs for TSS, TS, TA, VC, RS, and NRS

RNA-sequencing reads for ‘Allahabad Safeda’ and Purple Guava mixed fruit tissue and 3 days’ ripe fruit tissue were mapped to the draft genome assembly of guava using bowtie2 (Thakur et al., 2021). Several of the 58 QTLs identified for TSS, TS, RS, NRS, TA, and VC were found in the vicinity of annotated genes. The number of reads mapping to expressed genes in ‘Allahabad Safeda’ and Purple Guava were converted into log scale and are presented in the form of heat map.

## Results

**Figure 2:**
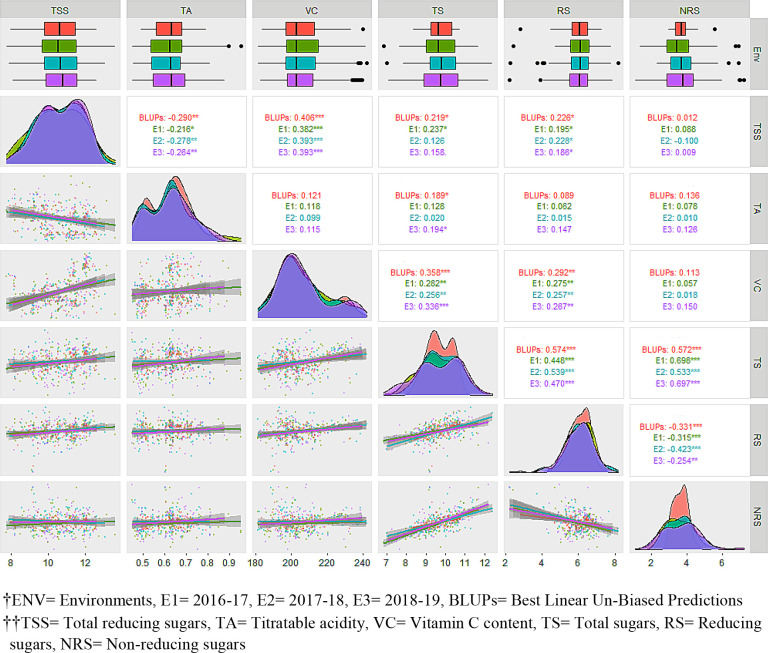
Evaluation of F_1_ hybrids of guava across three environments along with best linear unbiased predictions (BLUPs) for fruit physio-chemical traits.ENV, environment; E1 , 2016–17; E2 , 2017–18; E3 , 2018–19; BLUP, best linear unbiased prediction; TSS, total reducing sugars; TA, titratable acidity; VC, vitamin C content; TS, total sugars; RS, reducing sugars; NRS, non-reducing sugars.

Evaluation of the fruit physio-chemical traits of parents ‘Allahabad Safeda’ (AS) and Purple Guava (PG) differed markedly for fruit bio-chemical traits, and showed stability for these traits across the different environments (**Table 1**). To enhance the accuracy and mapping of stable QTLs across the different environments, linear mixed effects models were used to obtain BLUPs (genotypes as random effects) of fruit quality traits, accounting for genotype by environment interaction (G × E) effect. Among 125 F_1_ seedlings, 114 seedlings produced sufficient fruits for evaluation. The F_1_ population shows significant variation for fruit-quality traits across the different environments [BLUPs, 2016–17 (E1), 2017–18 (E2), and 2018–19 (E3)]. Although the mean values of TSS, TA, VC, TS, RS, and NRS content in F_1_ seedlings varied to some extent from year to year, normal distributions were observed in the studied period. Across the different environments, overall mean values of TSS, TA, and VC were highest in E3. Each phenotypic trait showed a little deviation from their normal data distribution across the studied environments (**Figure 2**), showing minute G × E effects suggestive of their polygenic nature and quantitative inheritance.

**Table 1:** Descriptive statistics of the physio-chemical traits of parents and the F_1_ population.

Trait	ENV	AS	PG	F_1_ population					
				Population	Mean	SD	CV	Skewness	Kurtosis
TSS (°B)	BLUPs	11.39	09.21	7.97–12.63	10.61	1.14	0.11	–0.22	–0.93
	E1	11.39	09.13	7.73–13.62	10.53	1.31	0.12	–0.08	–0.80
	E2	11.40	09.18	7.87–13.06	10.65	1.18	0.11	–0.17	–0.88
	E3	11.43	09.21	8.08–12.60	10.67	1.14	0.11	–0.29	–0.90
TA (%)	BLUPs	00.66	00.61	0.49–0.79	0.62	0.08	0.13	–0.10	–0.97
	E1	00.63	00.61	0.45–0.95	0.63	0.11	0.17	0.69	0.69
	E2	00.66	00.60	0.45–0.81	0.61	0.09	0.15	0.11	–0.76
	E3	00.70	00.60	0.45–0.88	0.63	0.10	0.16	0.17	–0.63
VC (mg/100 g)	BLUPs	224.94	191.01	183.69–239.99	206.21	13.22	0.06	0.66	–0.46
	E1	220.45	190.07	181.46–241.57	206.17	13.64	0.07	0.51	–0.53
	E2	223.81	188.76	181.32–242.20	206.31	14.21	0.07	0.64	–0.28
	E3	231.86	193.17	182.86–239.31	206.61	13.99	0.07	0.77	–0.21
TS (%)	BLUPs	10.32	09.33	8.34–10.76	9.69	0.62	0.06	–0.11	–1.01
	E1	10.26	09.19	6.85–11.67	9.62	1.00	0.10	–0.18	–0.61
	E2	10.43	09.21	7.02–12.41	9.80	0.93	0.10	–0.07	–0.13
	E3	10.91	09.22	7.09–12.20	9.67	1.12	0.12	–0.26	–0.73
RS (%)	BLUPs	06.46	02.82	2.82–7.28	6.03	0.62	0.10	–1.41	5.01
	E1	06.37	02.43	2.43–7.81	6.06	0.74	0.12	–1.00	3.91
	E2	06.54	02.28	2.28–8.20	6.03	0.87	0.14	–0.81	2.52
	E3	06.66	02.21	2.21–7.90	5.97	0.82	0.14	–0.95	2.85
NRS (%)	BLUPs	03.87	05.59	2.98–5.59	3.67	0.43	0.12	0.79	2.06
	E1	03.89	06.77	1.36–6.94	3.56	0.96	0.27	0.76	1.26
	E2	03.89	06.94	2.31–6.94	3.76	0.87	0.23	0.73	0.83
	E3	04.25	07.01	1.10–7.25	3.72	1.08	0.29	0.57	0.90

TSS, total reducing sugars; TA, Titratable acidity; VC, vitamin C; TS, total sugars; RS, Reducing sugars; NRS, Non-reducing sugars; ENV, Environments; E1 , 2016–17; E2 , 2017–18; E3 , 2018–19; BLUP, best linear unbiased prediction; SD, standard deviation; CV, coefficient of variation significant at α< 0.05.

### Estimates of coefficients of variation, heritability, and genetic advance

The genetic variability parameters genotypic coefficient of variance (GCV) and phenotypic coefficient of variance (PCV) are characterized as high at >20%, moderate at 10%–20%, and low at 0%–10%. Moderate GCV (16.13%) and PCV (19.59%) values were recorded for TA and NRS, respectively (**Table 2**), whereas lower GCV and PCV values were recorded for VC and TS. Environmental coefficient of variance (ECV) for all the traits was less than 10%, except for a higher value observed for NRS (21.63%). High heritability was observed for TSS (97.00%), TA (88.00%), VC (98.00%), and RS (86.00%), whereas moderate-to-high heritability was observed for TS (75.00%) and NRS (60.00%). Interestingly, TA (31.17%), TSS (22.22%), RS (21.25%), and NRS (24.21%) exhibited high GAM values, whereas VC (13.23%) and TS (13.28%) exhibited moderate GAM values.

**Table 2:** Descriptive statistics and estimates of genetic variability parameters of fruit physio-chemical traits among F_1_ hybrids of guava across different environments.

Trait	AS	PG	Variation in the F_1_ population							
	Mean	Mean	Mean	LSD (%)	CV (%)	GCV (%)	PCV (%)	ECV (%)	h^2^ (%)	GAM (%)
TSS (°B)	11.40	9.18	10.61	0.54	3.11	10.95	11.12	3.13	97.00	22.22
TA (%)	0.66	0.61	0.62	0.08	8.77	16.13	17.19	0.00	88.00	31.17
VC (mg/g)	225.26	190.75	206.21	5.72	1.70	6.49	6.56	1.70	98.00	13.23
TS (%)	10.48	9.24	9.69	1.02	7.49	7.44	8.59	7.51	75.00	13.28
RS (%)	6.51	2.43	6.03	0.70	7.65	11.12	12.00	7.60	86.00	21.25
NRS (%)	3.98	6.58	3.67	1.00	21.70	15.17	19.59	21.63	60.00	24.21

TSS, total reducing sugars; TA, Titratable acidity; VC, vitamin C; TS, total sugars; RS, Reducing sugars; NRS, Non-reducing sugars; GCV, genotypic coefficient of variance; PCV , phenotypic coefficient of variance; ECV , residual/environmental coefficient of variance; h^2^ , heritability broad sense; GAM , genetic advance as percent of mean; CV , coefficient of variation significant at α< 0.05 (CV); LSD, least significant difference significant at α< 0.05.

### Pairwise correlation and regression analysis

Pearson’s pairwise correlation was studied to identify the degree of correlation among fruit quality traits (**Figure 2**). There was a highly significant negative correlation between TSS and TA for BLUPs (–0.290), E1 (–0.216), E2 (–0.278), and E3 (–0.264), whereas a significant positive correlation was obtained between TSS and VC across the studied environments, viz., E1 (0.406), E2 (0.382), E3 (0.393), and BLUPs (0.393). The sugar components, i.e., TS and RS, showed a significant positive correlation with TSS. There was a highly significant negative correlation between RS and NRS but a positive correlation between TS and RS. TA showed significant correlation with TS for BLUPs (0.189) and E3 (0.194). Box-plots of the adjusted means of the fruit bio-chemical traits across the different environments are presented in **Figure 2**. Most of the traits appear to be normally distributed; however, some trait–environment combinations show skewed distributions, as demonstrated by the lopsided boxplots. Prominent data skewness was observed for TS and NRS in the first studied environment (E1). The estimated distribution with BLUPs was in agreement with observed distributions in different environments for most of the traits. Sugar components, i.e., RS and NRS, showed completely normal distribution, whereas the rest of the traits showed bimodal data distribution across the different environments. TSS in different environments, including BLUPs, shows a slight highest peak between 10% to 12% and lowest peak between 8% to 10%. Furthermore, the regression analysis shows that the TSS has negative association with TA and positive association with VC, TS, and RS. TSS has a somewhat linear relationship with NRS, as is also evident from the correlation analysis (**Figure 2**). There was a strong association among sugars (i.e., TS, RS, and NRS). Although RS has a strong negative relationship with NRS, a similar type of correlation between TSS and TA and between TSS and VC was observed.

### Principal component analysis, structural equation modeling for multivariate analysis, and clustering

**Figure 3:**
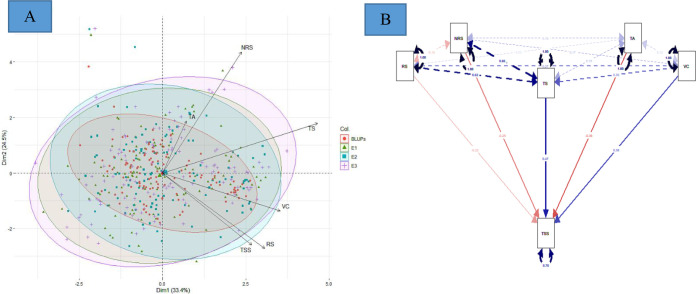
**(A)** Principal component analysis, and **(B)** multivariate analysis by structural equation modeling For **(A)**, red, green, blue, and purple represent eigen vectors for BLUPs, 2016–17 (E1), 2017–18 (E2), and 2018–19 (E3), respectively. For **(B)**, red and blue color represent negative and positive contribution, respectively.

The PCA offers details about traits by elucidating the population’s maximum variability across environments. The eigen vectors in the first two principal components (from PC1 to PC2) explained 57.9% of the total variability across the environments, showing a significant genotype by environment interaction (G × E). Overall, the first two dimensions of PCAs showed that TS was highly dependent on TA and NRS and least dependent on VC and RS (**Figure 3A**). Similarly, VC was highly dependent on RS. However, TSS was dependent on all the studied traits, with the most dependent variables being VC and RS. The principal component analysis reveals that the TSS was dependent on all the studied traits to different extents. SEM was performed to elucidate the direct or indirect variables that determined the TSS. TA, RS, and NRS had a small contribution toward TSS, while VC and TS were the major direct contributors to TSS, with a positive effect on TSS. Conversely, TA, RS, and NRS had negative effects on TSS (**Figure 3B**).

A total of 114 hybrids were grouped into four clusters based on fruit physico-chemical traits. A highly significant difference (*p*<0.001) was observed among inter-clusters of F_1_ individuals. We observed noticeable variability in the progeny for fruit quality traits. In total, 26.31% of descendants were found in Cluster-1 (female parent with superior fruit quality) and 38.60% in Cluster-2 (male parent) (**Table 3**). Interestingly, Cluster-4 was characterized by 18 descendants that had transgressive values compared to better parent for TSS and TS. Also, Cluster-1 exhibited higher VC and Cluster-2 had higher TA.

**Table 3:** Phenotypic clustering of 114 F_1_ individuals along with their respective parents for fruit physio-chemical traits.

Cluster	N	TSS (°B)	TA (%)	VC (mg/g)	TS (%)	RS (%)	NRS (%)
1^*^	30	10.62 ± 1.26^b^	0.59 ± 0.23^d^	208.61 ± 2.56^a^	9.69 ± 1.09^b^	5.84 ± 1.21^d^	3.83 ± 0.28^a^
2^**^	44	9.36 ± 1.46^d^	0.65 ± 0.21^a^	205.31 ± 3.25^d^	9.44 ± 2.01^d^	6.21 ± 1.33^b^	3.26 ± 0.58^c^
3	24	10.02 ± 1.35^c^	0.63 ± 0.05^b^	205.68 ± 1.69^c^	9.62 ± 1.33^c^	6.51 ± 0.89^a^	3.17 ± 1.23^d^
4^***^	18	11.08 ± 1.21^a^	0.60 ± 0.14^c^	206.62 ± 4.21^b^	9.78 ± 1.01^a^	5.99 ± 0.99^c^	3.79 ± 0.98^b^
*p*		0.001	0.001	0.001	0.001	0.001	0.001

*Allahabad Safeda, **Purple Guava, *** Transgressive segregants.

TSS, total reducing sugars; TA, titratable acidity; VC, vitamin C; TS, total sugars; RS, reducing sugars; NRS, non-reducing sugars. Superscripted alphabets (a-d) denotes the statistical significance between clusters (at p <0.001).

### Construction of linkage map

**Figure 4:**
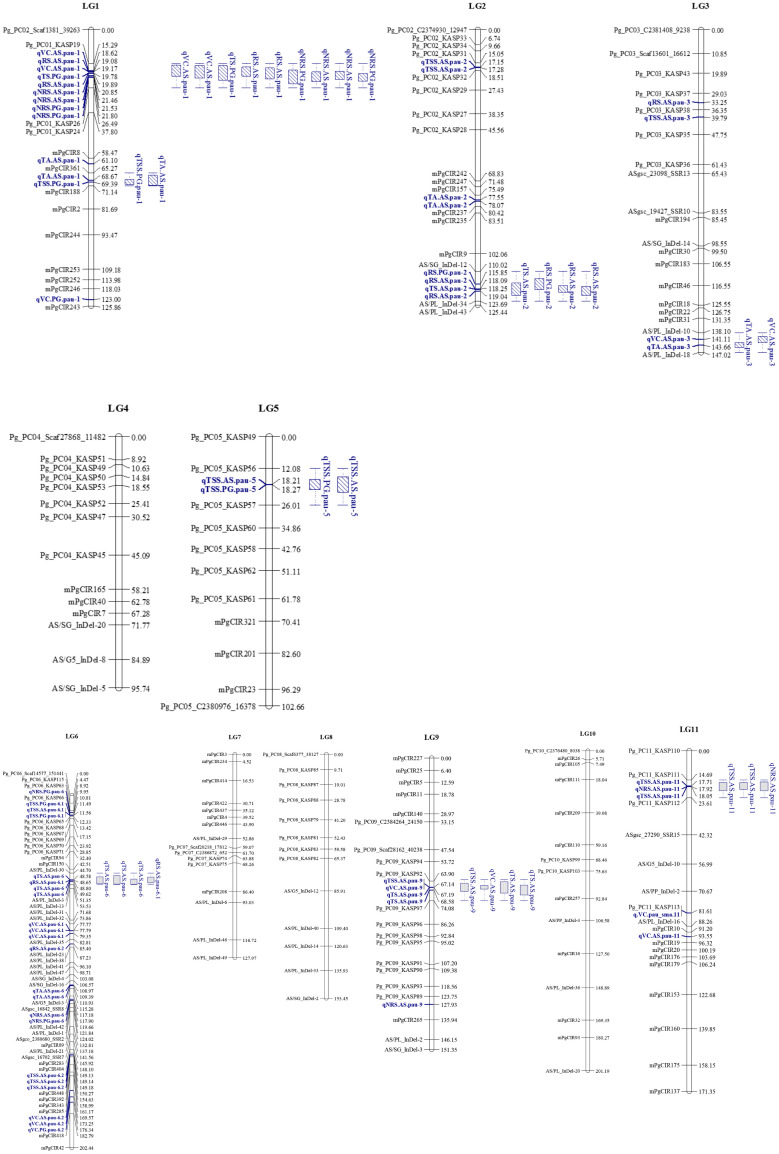
Genetic linkage groups (LGs) LG1 to LG5 of ‘Allahabad Safeda’ (AS) and Purple Guava (PG), and significant quantitative trait loci (QTLs) identified through the BIP (biparental populations) module of the composite interval mapping (CIM) algorithm. Blue color indicates QTLs, while blue boxes (bar graph) indicate the QTL clusters.

**Figure 5:**
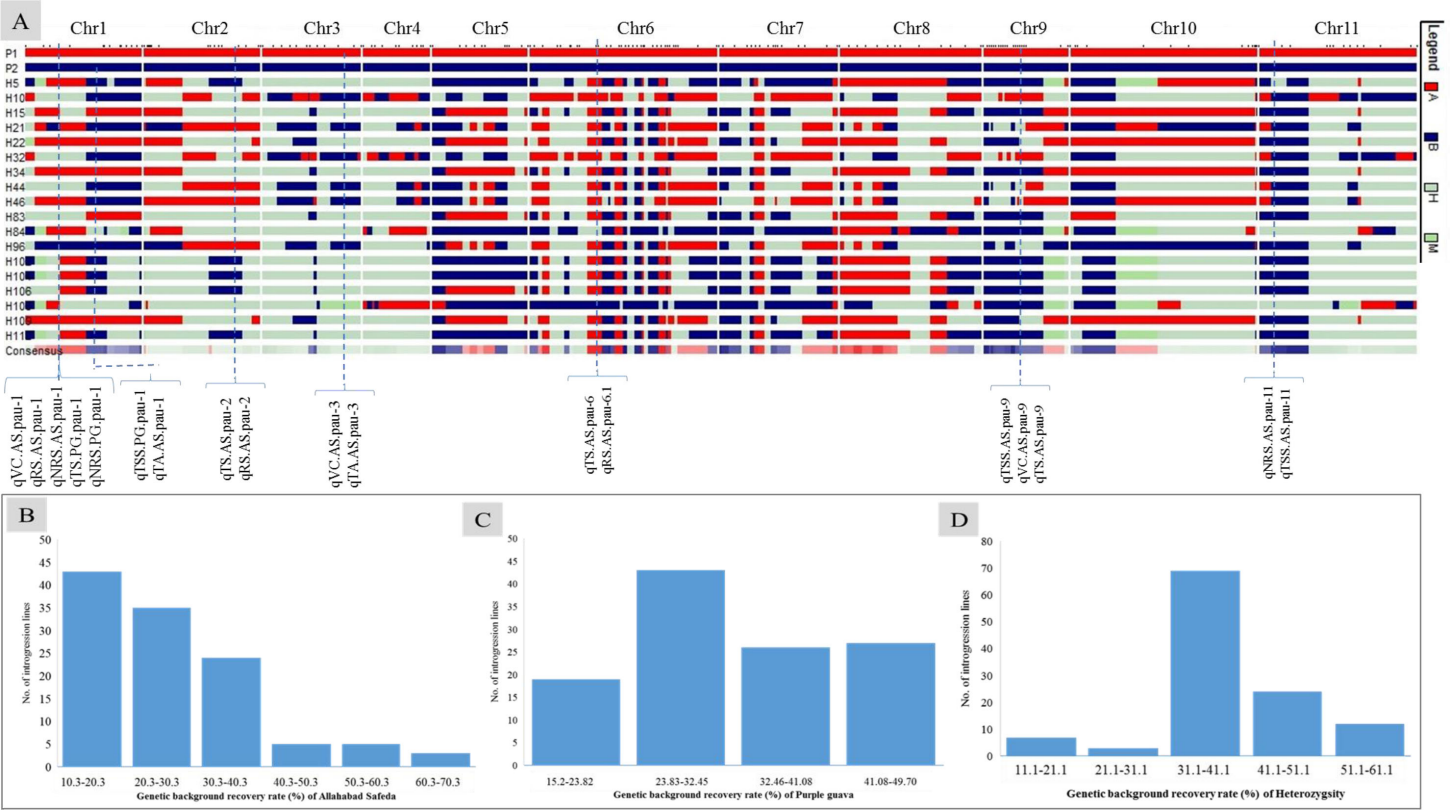
Genotypic data of 195 polymorphic markers used to generate **(A)** a graphical genotype pattern for the 18 transgressive guava hybrids lines, **(B)** the genetic background recovery rate of the female parent ‘Allahabad Safeda’ (AS), **(C)** the genetic background recovery rate of male parent Purple Guava (PG), and **(D)** residual heterozygosity (%) Red indicates presence of the female-specific allele (A: recipient parent), blue indicates the presence of the male-specific allele (B: Donor parent), gray indicates heterogeneous introgressions, and green indicates missing data.

We screened 328 SSR, SSR/InDels, and EST-SNP/KASP markers for a parental polymorphism survey of ‘AS’ and ‘PG’, revealing a polymorphism rate of 64.02% (i.e., in 210 out of 328 primer pairs). A linkage map consisting of 11 linkage groups (LGs) was constructed using 195 polymorphic markers (76 EST-KASP, 77 SSRs, and 42 EST-InDeLs), spanning a total length of 1,604.47 centimorgan (cM) and with an average distance of 8.80 cM between two adjacent markers (**Table 4**). LG4 was found to be the shortest linkage group at 95.74 cM (**Figure 4**), whereas LG6 was found to be the longest at 202.44 cM (**Figure 4**). The highest number of markers mapped (40) was on LG6 and the lowest number (11) was on LG5. The average length of LGs was 145.86 cM and the average interval distance ranged from 5.19 cM (LG6) to 14.37 cM (LG10). Fifteen SSR markers (4.57% of the total markers) showed a distorted segregation at *p* = 0.01. The genetic map covered 88.00% of the guava genome, with LG6 having the highest coverage at 95.00% and LG5 having the lowest coverage at 83.00% (**Table 4**). Genotyping followed by Graphical GenoTypes (GGT) analysis of 114 F_1_ individuals showed the highest proportion of PG introgressions (69.23%) on Chr6 and the lowest (7.50%) on Chr1. However, no introgression of the PG genome was observed on chromosomes 2, 7, and 9. The proportion of introgressed Purple Guava segments ranged from 15.2% to 49.7% in the F_1_ individuals. Our results showed that the average rate of genome background recovery was 26.5% for ‘Allahabad Safeda’ and 32.83% for Purple Guava. The F_1_ progeny exhibited 39.19% heterozygosity (**Figure 5**).

**Table 4:** Overview of genetic linkage map of ‘Allahabad Safeda’ (AS) and Purple Guava (PG).

LG	Total markers (*n*)	LG length (cM)^a^	cM/marker	Intervals (*n*)	Minimum intervals (cM)	Maximum intervals (cM)	Average intervals (cM)	Average Genome per LG ^b^	Genome coverage/ LG ^c^
1	13	125.86	9.68	12	4.07	20.67	10.48	146.82	0.86
2	17	125.44	7.38	16	1.75	23.27	7.84	141.12	0.89
3	19	147.02	7.74	18	0.95	18.12	8.17	163.36	0.90
4	14	95.74	6.83	13	1.71	14.57	7.36	110.46	0.87
5	11	102.66	9.33	10	6.37	13.93	10.27	123.2	0.83
6	40	202.44	5.06	37	0.00	21.62	5.19	212.82	0.95
7	16	127.97	7.99	15	2.18	23.69	8.51	144.99	0.88
8	13	153.45	11.80	12	5.79	23.49	12.79	179.03	0.86
9	20	151.35	7.57	19	2.18	14.39	7.96	167.27	0.90
10	15	201.19	13.41	14	1.78	21.39	14.37	229.93	0.88
11	17	171.35	10.08	16	2.55	18.71	10.70	192.75	0.89
Total	195	1,604.47	–	29.33	–	–	–	–	0.88
Minimum	11	95.74	5.06	10	0	13.93	5.19	–	–
Average	17.72	145.86	8.80	16.54	2.66	19.44	9.42	–	–
Maximum	40	202.44	13.41	37	6.37	23.69	14.37	–	–

a Length of LG constructed through QTLIci mapping.

b Length of LG estimated by the method of Fishman et al. (2001) and method 4 of Chakravarti et al. (1991).

c Calculated by dividing the observed LG length by the estimated genome length of the corresponding LG.

LG, linkage group.

### QTL mapping analysis of phenotypic data in individual environments and BLUP-values

Using the BIP (biparental populations) module of the ICIM-ADD algorithm, the phenotypic values of the three different environments and the BLUP values were used for QTL mapping. A total of 58 QTLs (16 in BLUP, 14 in E1, 12 in E2, and 16 in E3) associated with fruit-quality traits were identified, including 15 for TSS, 7 for TA, 12 for VC, 7 for TS, 8 for RS, and 9 for NRS (**Table 5**). These QTLs were mainly distributed on eight chromosomes with the most QTLs (i.e., 44) having beneficial alleles derived from the female parent ‘Allahabad Safeda’ (additive effect > 0), while the other 14 QTLs possessed alleles derived from the male parent Purple Guava. A maximum of 22 QTLs were detected on chromosome 6 and a minimum of two QTLs mapped on chromosome 5. These QTLs explained approximately 10.58%–17.85% of the phenotypic variation, with the LOD score ranging from 3.01 to 5.96. The highest absolute additive effect value was 1.34 and was observed for the TSS trait (qTSS.AS.pau-6.2).

**Table 5:** Summary of the significant quantitative trait loci (QTLs) detected through a composite interval mapping (CIM) algorithm in the mapping population derived from the cross of guava cv. 'Allahabad Safeda' and 'Purple Guava'.

Trait	ENV	Chr	QTL^a^	Position	Marker interval	Interval (cM)	LOD^b^	PVE^c^	ADD	Kruskal–Wallis analysis^d^
TSS	B	2	qTSS.AS.pau-2 ^#^	17.28	Pg_PC02_KASP31–Pg_PC02_KASP32	16.08–18.48	3.13	11.25	1.20	****
	B	5	qTSS.PG.pau-5 ^#^	18.27	Pg_PC05_KASP56–Pg_PC05_KASP57	16.28–20.25	3.07	13.64	–0.07	****
	B	6	qTSS.PG.pau-6.1 ^#^	11.56	Pg_PC06_KASP66–Pg_PC06_KASP65	10.99–22.12	3.01	13.64	–0.17	***
	B	6	qTSS.AS.pau-6.2 ^#^	149.18	mPgCIR404–mPgCIR392	148.15–150.21	5.96	13.91	1.34	****
	B	11	qTSS.AS.pau-11 ^#^	18.05	Pg_PC11_KASP111–Pg_PC11_KASP112	15.99–20.10	3.56	12.01	0.06	***
	E1	3	qTSS.AS.pau-3	39.79	Pg_PC03_KASP38–Pg_PC03_KASP35	37.02–42.56	3.13	13.77	1.11	****
	E1	6	qTSS.PG.pau-6.1 ^#^	11.49	Pg_PC06_KASP66–Pg_PC06_KASP65	10.96–12.01	3.02	12.58	–0.12	****
	E1	6	qTSS.AS.pau-6.2 ^#^	149.14	mPgCIR404 –mPgCIR392	148.19–150.09	4.12	14.15	1.33	**
	E2	6	qTSS.AS.pau-6.1 ^#^	11.56	Pg_PC06_KASP66–Pg_PC06_KASP65	10.86–12.26	3.25	15.23	1.26	**
	E2	6	qTSS.AS.pau-6.2 ^#^	149.13	mPgCIR404 –mPgCIR392	148.25–150.01	3.11	10.58	1.16	***
	E2	9	qTSS.AS.pau-9	67.14	Pg_PC09_KASP92–Pg_PC09_KASP97	65.03–69.25	3.28	12.98	1.01	***
	E3	2	qTSS.AS.pau-2 ^#^	17.15	Pg_PC02_KASP31–Pg_PC02_KASP32	16.00–18.30	3.69	13.33	0.99	**
	E3	1	qTSS.PG.pau-1	69.39	mPgCIR361–mPgCIR188	68.32–70.45	4.01	14.15	–0.29	****
	E3	5	qTSS.AS.pau-5 ^#^	18.21	Pg_PC05_KASP56–Pg_PC05_KASP57	15.26–21.16	3.03	13.74	1.25	****
	E3	11	qTSS.AS.pau-11 ^#^	17.71	Pg_PC11_KASP111–Pg_PC11_KASP112	16.02–19.40	4.33	13.62	1.08	***
TA	B	2	qTA.AS.pau-2 ^#^	77.55	mPgCIR157 - mPgCIR237	75.53–79.56	5.91	13.91	1.10	****
	B	6	qTA.AS.pau-6 ^#^	108.97	AS/SG_InDel-16–AS/G5_InDel-3	107.98–109.96	3.20	13.27	1.32	***
	E1	1	qTA.AS.pau-1	61.10	mPgCIR8–mPgCIR361	59.16–63.03	3.39	13.25	1.15	***
	E1	3	qTA.AS.pau-3	143.66	AS/PL_InDel-10–AS/PL_InDel-18	142.56–144.75	3.05	13.19	0.87	**
	E2	2	qTA.AS.pau-2 ^#^	78.07	mPgCIR157 –mPgCIR237	75.98–80.15	3.01	12.56	0.52	****
	E3	1	qTA.AS.pau-1	68.67	mPgCIR361- mPgCIR188	66.31–71.03	3.18	12.78	0.19	***
	E3	6	qTA.AS.pau-6 ^#^	109.39	AS/SG_InDel-16–AS/G5_InDel-3	108.54–110.24	3.69	14.05	–0.22	****
VC	B	6	qVC.AS.pau-6.1 ^#^	77.79	AS/PL_InDel-32–AS/PL_InDel-35	75.42–80.16	4.01	12.69	1.00	***
	B	6	qVC.PG.pau-6.2 ^#^	176.34	mPgCIR285–mPgCIR418	171.15–181.52	3.81	12.88	–0.27	****
	B	11	qVC.AS.pau-11	93.55	mPgCIR10–mPgCIR19	92.09–95.01	4.02	13.35	1.10	*****
	E1	1	qVC.PG.pau-1	123.00	mPgCIR246–mPgCIR243	121.02–124.98	3.32	13.47	–0.11	***
	E1	3	qVC.AS.pau-3	141.11	AS/PL_InDel-10–AS/PL_InDel-18	139.63–142.58	3.25	12.24	1.16	***
	E1	9	qVC.AS.pau-9	67.14	Pg_PC09_KASP92–Pg_PC09_KASP97	66.25–68.03	3.41	12.98	0.87	****
	E2	1	qVC.AS.pau-1 ^#^	18.62	Pg_PC01_KASP19–Pg_PC01_KASP26	16.08–21.15	3.78	13.33	0.96	****
	E2	6	qVC.AS.pau-6.1 ^#^	79.35	AS/PL_InDel-32–AS/PL_InDel-35	76.54–82.15	4.13	12.05	1.10	****
	E2	6	qVC.AS.pau-6.2 ^#^	173.25	mPgCIR285 –mPgCIR418	165.24–181.26	3.08	13.54	0.58	**
	E3	1	qVC.AS.pau-1 ^#^	19.17	Pg_PC01_KASP19–Pg_PC01_KASP26	16.32–22.02	3.69	14.42	1.00	***
	E3	6	qVC.AS.pau-6.1 ^#^	77.77	AS/PL_InDel-32–AS/PL_InDel-35	74.12–81.42	4.12	13.54	–0.31	****
	E3	6	qVC.AS.pau-6.2 ^#^	169.57	mPgCIR285 –mPgCIR418	162.89–176.24	3.89	14.21	0.11	****
TS	B	6	qTS.AS.pau-6 ^#^	48.80	AS/PL_InDel-30–AS/PL_InDel-3	46.85–50.74	4.11	13.18	0.78	****
	B	9	qTS.AS.pau-9 ^#^	67.19	Pg_PC09_KASP92–Pg_PC09_KASP97	64.25–70.12	4.15	13.09	0.65	****
	E1	1	qTS.PG.pau-1	19.78	Pg_PC01_KASP19–Pg_PC01_KASP26	16.45–23.10	4.47	12.41	–0.21	***
	E1	2	qTS.AS.pau-2	118.25	AS/SG_InDel-12–AS/PL_InDel-34	115.37–121.12	4.31	11.02	0.39	***
	E2	6	qTS.AS.pau-6 ^#^	48.58	AS/PL_InDel-30–AS/PL_InDel-3	46.12–51.03	3.65	11.48	0.87	****
	E3	6	qTS.AS.pau-6 ^#^	49.62	AS/PL_InDel-30–AS/PL_InDel-3	48.12–51.12	3.41	12.29	1.21	***
	E3	9	qTS.AS.pau-9 ^#^	68.58	Pg_PC09_KASP92–Pg_PC09_KASP97	66.01–71.15	3.27	12.88	0.49	****
RS	B	2	qRS.PG.pau-2 ^#^	115.85	AS/SG_InDel-12–AS/PL_InDel-34	113.25–118.45	3.02	12.20	–0.13	
	B	3	qRS.AS.pau-3	33.25	Pg_PC03_KASP37–Pg_PC03_KASP38	30.18–36.32	4.29	14.56	0.16	
	E1	1	qRS.AS.pau-1 ^#^	19.08	Pg_PC01_KASP19–Pg_PC01_KASP26	17.01–21.15	3.33	12.25	1.05	***
	E1	2	qRS.AS.pau-2 ^#^	118.09	AS/SG_InDel-12–AS/PL_InDel-34	116.53–119.65	3.41	12.18	0.74	***
	E2	1	qRS.AS.pau-1 ^#^	19.89	Pg_PC01_KASP19–Pg_PC01_KASP26	17.42–22.36	4.47	11.56	0.69	****
	E2	2	qRS.AS.pau-2 ^#^	119.04	AS/SG_InDel-12–AS/PL_InDel-34	117.02–121.05	4.03	11.33	1.01	****
	E3	6	qRS.AS.pau-6.1	48.65	AS/PL_InDel-30–AS/PL_InDel-3	47.01–50.28	4.13	11.48	1.12	***
	E3	6	qRS.AS.pau-6.2	85.40	AS/PL_InDel-35–AS/PL_InDel-23	83.65–87.14	3.69	13.52	1.29	**
NRS	B	1	qNRS.PG.pau-1 ^#^	21.53	Pg_PC01_KASP19–Pg_PC01_KASP26	18.47–24.58	4.37	17.77	–0.18	****
	B	6	qNRS.AS.pau-6 ^#^	117.18	ASgsc_16842_SSR8–AS/PL_InDel-42	115.96–118.39	4.57	15.33	0.24	****
	E1	1	qNRS.AS.pau-1 ^#^	21.46	Pg_PC01_KASP19–Pg_PC01_KASP26	19.26–23.65	3.85	16.85	0.84	***
	E1	6	qNRS.PG.pau-6 ^#^	117.90	ASgsc_16842_SSR8–AS/PL_InDel-42	116.25–119.55	3.47	16.96	–0.15	****
	E2	1	qNRS.AS.pau-1 ^#^	20.85	Pg_PC01_KASP19–Pg_PC01_KASP26	19.01–22.69	3.61	17.05	0.87	****
	E2	9	qNRS.AS.pau-9	127.93	Pg_PC09_KASP89–mPgCIR265	125.64–130.21	4.15	17.85	1.12	***
	E3	1	qNRS.PG.pau-1 ^#^	21.80	Pg_PC01_KASP19–Pg_PC01_KASP26	20.03–23.56	4.18	16.98	–0.19	***
	E3	6	qNRS.PG.pau-6	9.95	Pg_PC06_KASP63–Pg_PC06_KASP66	9.16–10.74	3.65	15.21	–0.21	****
	E3	11	qNRS.AS.pau-11	17.92	Pg_PC11_KASP111–Pg_PC11_KASP112	15.69–20.14	3.12	14.24	0.41	****

TSS, total reducing sugars; TA, titratable acidity; VC, vitamin C content; TS, total sugars; RS, reducing sugars; NRS, non-reducing sugars; ENV, Environments; E1 , 2016–17; E2 , 2017–18; E3 , 2018–19; BLUPs, best linear unbiased predictions; Chr, chromosome; cM, centimorgan; ADD, additive effect.

a QTLs are named using “q” as a prefix followed by abbreviated names of the measured traits, the location, alleles-contributing parent, and a chromosome number

b LOD= logarithm of odds (generally ≥ 3).

c The percentage of total phenotypic variance explained by the QTL.

d Significance levels **0.001; ***0.0005; ****0.0001.

^#^Stable QTLs = detected across the multiple environments within the specified overlapping marker intervals. ‘Allahabad Safeda’ and purple guava.

### Stability of QTLs over multiple environments

The BLUP values effectively reduced the impact of environmental differences. Considering QTLs detected using BLUP datasets, 13 out of 16 were repeatedly detected in multiple environments or across different environments, while the others could be regarded as environment specific. Five QTLs for TSS were identified using BLUP data located on LG2, LG5, LG6, and LG11 (**Table 5**). qTSS.PG.pau-6.1 flanked by Pg_PC06_KASP66 and Pg_PC06_KASP65 was detected in two individual environments, E1 and E2, with 12.58%–15.23% of the total PVE. In addition, qTSS.AS.pau-6.2 flanked by mPgCIR404 and mPgCIR392 was detected in E1 and E2 with LOD scores of 4.12 and 3.25, respectively. Similarly, QTL qTSS.AS.pau-2, located on chromosome 2 and flanked by Pg_PC02_KASP31 and Pg_PC02_KASP32, was detected in environment E3 with a LOD score of 3.69 and 13.33% PVE. Moreover, two QTLs associated with TSS, qTSS.PG.pau-5 and qTSS.AS.pau-11, could be detected in E3, and its position was localized within 14.01–21.41 cM and 15.99–20.10 cM marker intervals, respectively. The QTL analysis revealed that TA was controlled by seven QTLs. Among them, two loci were repeatedly detected in multiple environments. qTA.AS.pau-2, located on chromosome 2, was co-detected in the E2 and BLUP datasets, explaining 12.56% and 13.91% of the phenotypic variation, respectively. QTL qTA.AS.pau-6 was identified using the BLUP dataset and was co-detected in E3 with a relatively high PVE (14.05%). From the 12 QTLs identified for vitamin-C content, we found two QTLs, qVC.AS.pau-6.1 and qVC.PG.pau-6.2, on LG6 using BLUP datasets, which were simultaneously co-detected in E2 and E3, explaining 12.05–13.54% and 13.54–14.21% of phenotypic variation, respectively. q.TS.AS.pau-6 and qTS.AS.pau-9 were repeatedly identified in at least two datasets within the marker intervals of AS/PL_InDel-30-AS/PL_InDel-3 and Pg_PC09_KASP92-Pg_PC09_KASP97. Notably, q.TS.AS.pau-6 had the greatest contribution for total sugars and explained 13.18% of variation based on BLUP values. A single QTL, qRS.AS.pau-2, accounted for 11.33%, 12.18%, and 12.20% of phenotypic variation in the E1, E2, and BLUP datasets, respectively. q.NRS.PG.pau-1, detected in all the studied environments including BLUP, was identified within the marker interval of Pg_PC01_KASP19–Pg_PC01_KASP26, explaining 16.85%–17.77% of the phenotypic variation. Thus, the QTL *q.NRS.PG.pau-1*, with >15% PVE in all environments, could be a focus for further in-depth study of the non-reducing sugars trait in guava.

### QTL clusters for guava fruit quality

Considering QTLs with overlapping confidence intervals as QTL clusters, we identified five QTL clusters related to different fruit-quality traits (**Table 6**). The mPgCIR361–mPgCIR188 interval on LG1 was involved in the control of TSS (qTSS.PG.pau-1) and TA (qTA.AS.pau-1) (**Figure 4**). Nine QTLs controlling VC, TS, RS, and NRS were detected in the partially overlapping interval of Pg_PC01_KASP19–Pg_PC01_KASP26 on LG1. QTL qNRS.PG.pau-1 was detected in multiple environments with a relatively high PVE of 17.77%. This indicates that variation in NRS has a large effect in special environment. Furthermore, this QTL exhibited negative additive effects, indicating that the NRS-increasing effect of this locus came from the male parent PG. The interval AS/SG_InDel-12–AS/PL_InDel-34 on LG2 was involved in total sugars (qTS.AS.pau-2) and reducing sugars (qRS.AS.pau-2). The interval AS/PL_InDel-30–AS/PL_InDel-3 on LG6 harbored three QTLs influencing total sugars and reducing sugars. The interval Pg_PC09_KASP92–Pg_PC09_KASP97 on LG9 harbored four QTLs (qTSS.AS.pau-9, qVC.AS.pau-9, qTS.AS.pau-9, and qTS.AS.pau-9) that influenced vitamin C and total sugars. The results suggest that some genes involved in non-reducing sugars (qNRS.AS.pau-11) on LG11 also affect TSS, as qTSS.AS.pau-11 was identified by BLUP. However, these genes have a relatively larger impact on NRS (PVE = 15.21%) and TSS (PVE = 15.99%). The phenomenon of QTL clusters could explain the very high correlation of traits and the linkage drag, which often hinder breeding programs for improving fruit quality. Thus, molecular assisted selection (MAS), could be used effectively to improve the linked fruit-quality traits mapped over the overlapping intervals.

**Table 6:** Quantitative trait locus (QTL) clusters for fruit quality traits in the guava population (‘Allahabad Safeda’ × Purple Guava).

Cluster	QTL	Chr	Position	Nearest marker interval	EE (*n*)
C1	qTSS.PG.pau-1	1	69.39	mPgCIR361–mPgCIR188	1
	qTA.AS.pau-1	1	68.67		1
C2	qVC.AS.pau-1	1	18.62, 19.17	Pg_PC01_KASP19–Pg_PC01_KASP26	2
	qTS.PG.pau-1	1	19.78		1
	qRS.AS.pau-1	1	19.08, 19.89		2
	qNRS.AS.pau-1	1	20.85, 21.46		2
	qNRS.PG.pau-1	1	21.53, 21.80		2
C3	qTS.AS.pau-2	2	118.25	AS/SG_InDel-12–AS/PL_InDel-34	1
	qRS.PG.pau-2	2	115.85		1
	qRS.AS.pau-2	2	118.09, 119.04		2
C4	qTA.AS.pau-3	3	143.66	AS/PL_InDel-10–AS/PL_InDel-18	1
	qVC.AS.pau-3	3	141.11		1
C5	qTS.AS.pau-6	6	48.58, 48.80, 49.62	AS/PL_InDel-30–AS/PL_InDel-3	3
	qRS.AS.pau-6.1	6	48.65		1
C6	qTSS.AS.pau-9	9	67.14	Pg_PC09_KASP92–Pg_PC09_KASP97	1
	qVC.AS.pau-9	9	67.14		1
	qTS.AS.pau-9	9	67.19, 68.58		2
C7	qTSS.AS.pau-11	11	17.71, 18.05	Pg_PC11_KASP111–Pg_PC11_KASP112	2
	qNRS.AS.pau-11	11	17.92		1

Chr, Chromosome; EE, environments of QTL expression; QTL , quantitative trait locus.

The QTLs controlling TSS, TS, RS, NRS, TA, and VC were found to be associated with 26 annotated genes in the draft genome assembly of guava (**Supplementary Figure 1**). Several of the genes were found to be overexpressed in PG fruit tissue, including BEACH (beige and Chediak Higashi) domain-containing protein C2-like controlling vesicle trafficking and protein sorting. Higher expression of serpin-ZX with protease inhibitor activities endorses the non-reducing sugars enrichment in Purple Guava.

## Discussion

### Phenotypic variations and genetic variability estimates

Fruit-quality attributes are the one of the vital characteristic traits for horticultural crops. The fruit biochemical traits, viz., TSS, TA, VC, and sugars, are critical determinants of the fruit quality of guava (Patel et al., 2014). The F_1_ population exhibited significant variations among fruit-quality traits and the female parent ‘Allahabad Safeda’ exhibited consistently higher values of TSS, TA, VC, and RS in all three environments. Conversely, the male parent Purple Guava was characterized by higher non-reducing sugars. The replicated phenotypic evaluations of these traits across the different environments enhances the precision and reliability of QTL mapping, especially for the traits with low heritability (Alimi et al., 2013). The presence of transgressive segregants is probably owing to the genotypic difference of parents influencing the segregation patterns in the offspring (Goulet et al., 2017; Koide et al., 2019). The descriptive statistics of TSS, TA, and VC observed were within the ranges of previous studies (Rodríguez et al., 2007; Patel et al., 2011). Similar findings have been reported for sugar content among different hybrids of guava (Singh, 2017; Singh, 2020). Comparative variability of fruit-quality traits is evaluated by determining the genotypic parameters (viz., GCV, PCV, h^2^, and GAM) and it becomes absolutely necessary to identify genotypes with higher genetic potential for their utilization in developing an efficient breeding strategy. Moderate PCV and GCV values were recorded for TSS, TA, RS, and NRS, suggesting a good amount of variability among hybrids for these traits. Conversely, TS has low GCV and PCV values, indicating a high influence of environmental conditions on these traits. However, a slight difference between PCV and GCV values for all the evaluated traits suggests that there was minimal influence of environments in the expression of these traits. Although GCV is helpful for the measurement of the presence of a high degree of genetic variation, the amount of heritable portion can only be determined with the help of heritability estimates and genetic gains (Rao and Rao, 2015). The heritability of a trait, as a proportion of the phenotypic variation that is attributed to genetic causes, has been a prime indicator of Heritability and helpful in taking decisions for the genetic improvement of economic traits (Narain, 2010). Heritability of a trait is a key component in determining GAM (Nyquist, 1991). Heritability along with genetic advance is more effective and reliable in predicting the best individuals (Panse, 1942; Johnson et al., 1955; Lerner, 1958). The studied fruit quality has high heritability and higher GAM, which indicate that the expression of these traits is governed by additive gene action and these characters can be easily improved by phenotypic selection methods.

### Correlation analysis

In fruit breeding, the degree of association of different traits has always been useful for selection of fruit-quality traits, especially when these follow quantitative inheritance and/or are influence by the environment. Breeders generally focus on the improvement of multiple traits, so the correlation analysis is mostly used for studying the existence of associations between different traits. Therefore, correlation studies play an important role in determining the most efficient breeding procedure. There were different degrees and magnitudes of correlation between the studied fruit bio-chemical traits. Phenotypic correlations between TSS, VC, TS, and RS were significantly positive, indicating the existence of shared genetic control between these traits. Conversely, there was a negative correlation of TSS with TA. Significantly positive correlations appeared between TSS and NRS in two or more environments. Rodríguez et al. (2007) reported a highly significant correlation of TSS with TA in three mapping populations of guava. Linear relationships with significant correlations are common among fruit-quality traits (Thimmappaiah et al., 1985; Rajan et al., 2005; Rajan et al., 2008; Singh, 2015) and our results reflect this. The lower variance for BLUPs than the different studied environments meant that BLUPs were able to reduce the environmental variance across the years to a great extent (**Figure 2**). The fruit bio-chemical traits’ distribution curves further agreed with this, showing better normal distribution. The highest peaks of TS, RS, and NRS were achieved with the predictor (BLUPs), indicating the potential of this breeding program. Similarly, the first two dimensions of principal components showed that BLUPs was able to explain the variance of studied traits across different environments. The PCA studies explain that TSS is strongly influenced by VC and RS, while NRS was more strongly associated with TA and TS (**Figure 3A**). Through a combined correlation and path analysis, we provided a first insight into the nature and magnitude of the complex interactions between fruit-quality traits of guava.

### Selection of excellent introgressed lines

Genotypic selection is very difficult with a large number of individuals (Noerwijati et al., 2021). Clustering (multivariate analysis) is an efficient tool for the genotypic selection process (Kozak et al., 2008) that classifies the phenotypic variations with the function of homogeneity within intra-clusters and heterogeneity among the inter-clusters (Oliveira et al., 2016). Significant intra-cluster variability (*p*< 0.01) was observed with respect to fruit-quality traits in guava. Cluster analysis is a complementary tool to PCA (El-Hashash, 2017). Hybridizations often produce transgressive segregating progenies in guava (Patel et al., 2007; Singh, 2020). Eighteen transgressive hybrids from cluster-4 with the best comprehensive TSS and TS phenotypes stable over all environments have set a milestone in strategic breeding for guava improvement.

### Linkage mapping and quantitative trait loci analysis

Guava has the potential for higher productivity and nutritional quality (Sanda et al., 2011). Several hybrids have been released recently that fulfill one of the breeding objectives for guava (Pandey et al., 2007; Singh, 2015; Singh, 2020), but there is still a dearth of perfected cultivars with traits of commercial importance (Dinesh and Vasugi, 2010). Major hybridization limitations such as guava’s perennial nature, long juvenile phase, heterozygous nature, and epigynous floral structure limits the speed of genetic improvement (Pommer and Murakami, 2008). With the advancement of molecular markers and the concept of linkage mapping (Babu et al., 2004), speeded-up genetic improvement has been achieved in different fruit crops of a perennial nature (Mathiazhagan et al., 2021). Mostly, the microsatellite (SSR-based) markers have been applied for genetic map construction and the exploration of genomic regions of horticultural crops (Ahmad et al., 2020), but the molecular exploration of guava is still in its infancy owing to insufficient genomic resources (Nimisha et al., 2013; Padmakar et al., 2016). The first molecular linkage map in guava was established using AFLP markers (167 primers mapped onto 11 linkage groups) by Valdés-Infante et al. (2003). Only a few genetic maps, which predominantly use SSRs in combination with AFLP, RAPD, and SRAP markers, have been published (Rodríguez et al., 2007; Lepitre et al., 2010; Ritter et al., 2010; Padmakar et al., 2016; Sohi et al., 2022).

The present genetic map was made for 11 linkage groups, comprising 76 EST-KASP, 77 SSR, and 42 EST-InDeL markers, with a genome spanning 1,604.47 cM and an average distance of 8.80 cM between markers (**Table 4**). Relative to previously published genetic maps in guava, this is the first common genetic map based on SNPs and InDels markers with a wider marker density and greater genome-wide span. NGS-based technologies have resulted in the availability of large genomic resources and enriched the marker repository (Pérez-de-Castro et al., 2012). SNPs and InDels relatively pose more abundance, can be scored with high accuracy, are highly repeatable, and are spread over the entire genome (Liu B et al., 2013; Yamaki et al., 2013; Wu J et al., 2014s; Liu et al., 2015; Verma et al., 2015). Our study is the first of its kind to report the development of a linkage map using SNPs and InDels markers as well as the exploration of guava genomic regions. This linkage map has the smallest average marker intervals (9.42 cM) when compared with previous linkage maps in guava, making it ideal for genetic mapping of desirable traits. After the construction of the genetic linkage map, QTL analysis was performed to understand the genetic architecture of fruit physico-chemical-related traits. By ascertaining the number, relative positions, and effect of the markers underlying phenotypic variation, the QTL results open up new prospects for the implementation of molecular-assisted selection (MAS), which can ultimately accelerate and maximize genetic gains (Collard and Mackill, 2008). Despite the great advances in the genomics of horticultural crops, guava has received meagre attention with respect to the establishment of genotype–phenotype associations (Mittal et al., 2020). We previously developed a biparental mapping population of guava (‘Allahabad Safeda’ × Purple Guava) aimed at mapping leaf anthocyanin coloration (Sohi et al., 2022), and this trait shows segregation and normal distribution in the mapping population. Thus, the population should be useful for identifying QTLs for other important traits (Sohi et al., 2022).

The genetic factors for fruit-quality traits have long been a concern of fruit breeders. Molecular markers enhance the ability to determine the inheritance and parentage of specific genomic regions and to monitor the introgression of specific chromosomal segments that are linked to desirable traits in breeding lines (Vishwakarma et al., 2022). Graphical genotyping software programs, such as GGT (van Berloo, 2008), are very useful tools for selecting preferable progenies on the basis of their genotypic content. The number of markers tested on each chromosome of phenotypically selected transgressive segregants is shown in **Figure 5A**, along with the number and introgressed proportion/average recovery of ‘Allahabad Safeda’ (**Figure 5B**), Purple Guava (**Figure 5C**), and heterozygosity (**Figure 5D**). On the basis of GGT analysis, eight F_1_ transgressive progenies (H52, H90, H48, H40, H38, H81, H36, and H97) showing stable values of TSS (> 12.01 °B) and TS (> 9.88%) in four different environments cover more than 50% of the ‘Allahabad Safeda’ genome. Therefore, the F_1_ individuals from Cluster-4 could be useful in developing cultivars with high TSS and total sugar content.

Tremendous efforts have been made to identify QTLs for fruit-quality traits in different fruit crops (Wu J et al., 2014; Liu et al., 2016; Nantawan et al., 2019; Shi et al., 2020). However, limited efforts have been made in genetic dissection and QTL discovery for fruit quality in guava. The present genetic map of a cross between ‘Allahabad Safeda’ and Purple Guava has the highest marker density of any maps published so far. Most of the fruit-quality characteristics are of a quantitative nature, and it is very important to locate QTLs for fruit quality in guava and estimate their effects. The 195 markers cover the whole genome of guava, ensuring that all the introgressed segments are identified (**Figure 5A**). The effectiveness of markers associated with detected QTLs should be determined as the percentage of the explained genetic variance (Nishio et al., 2011). The introgressed segments were evaluated for favorable QTL alleles. Fruit quality is particularly influenced by the environmental conditions. Understanding the genetics of fruit physio-chemical characteristics related to fruit quality is crucial for guava breeding programs worldwide. To date, no such comprehensive research has been executed to identify genomic regions in the guava associated with these traits. We identified 58 QTLs located on eight chromosomes related to six fruit-quality traits with approximately 10.58%–17.85% PVE. Guava fruits are expected to have a sweet taste, and consumer acceptance is associated with the ripe soluble solids concentration reaching 10%–11%, a sugar–acid ratio of approximately 7:13, and total sugar content of approximately 7%–9%. TSS and sugar–acid blend are the central determinants of fruit quality. A total of 15 QTLs associated with TSS were detected on LG1, LG2, LG3, LG5, LG6, LG9, and LG11, whereas QTLs associated with fruit acidity (TA) have been mapped onto four LGs: LG1, LG2, LG3, and LG6. Rodríguez et al. (2007) reported two QTLs for TSS (PVE of approximately 7.9%–8.3%), two for vitamin C (PVE of approximately 5.4%–6.0%), and three for titratable acidity (PVE of approximately 8.3%–12.0%) located on LG5, LG6, LG7, and LG10. However, the PVEs in our study are higher than previous reports. Vitamin-C content in fruit may have health benefits as a source of antioxidants and higher sugar levels are a means of promoting the guava commercially. Nine QTLs associated with vitamin C mapped on LG1, LG3, LG6, and LG9 showed dominant inheritance and had PVEs between 12.05% and 14.21%. Our findings are consistent with the literature, as Ritter et al. (2010) reported two QTLs for acidity (approximately 7.2%–9.8% PVE), four QTLs for TSS (approximately 6.6%–10.9% PVE), and seven QTLs for vitamin C (approximately 4.9%–11.5% PVE) on LG1, LG2, LG4, LG6, LG7, LG8, and LG10. QTLs across different studies are not always the same due to differences in the genetic background of materials, environments, and cultivation methodologies. Therefore, QTL validation through replications over multiple environments and agroclimatic zones is crucial (Zhaoming et al., 2017).

The BLUP analysis combines the selection index and least square methods, which were proposed for animal breeding (Henderson, 1974). This method considers fixed environmental and random genetic effects at the same time, thus increasing the accuracy of the prediction in different years, environments, and generations (Piepho et al., 2008: Wang et al., 2016). This method is now widely used in genome-wide association studies (GWAS), genomic selection (GS), and QTL mapping. Out of the 16 QTLs identified using BLUP datasets, 13 QTLs were detected in multiple environments. These stable QTLs would be valuable for guava breeding and are suggestive of genetic interactions owing to the presence of similar genes and their expression pattern, exhibited in terms of phenotype. Furthermore, the use of BLUPs improved the QTL detection power by blurring the environmental variance. Indeed, several studies have demonstrated the effectiveness of using BLUPs for QTL localization in different fruit species such as apple (Segura et al., 2009), citrus (Khefifi et al., 2022), and grapevine (Doligez et al., 2013), where the experimental material had been phenotyped under different environments. The observed instability in common QTL detection might be due to a complex quantitative genetic model and different physiological mechanisms in the determination of different fruit-quality components in response to environmental variations between years (Fanizza et al., 2005). Thus, the identification of different QTLs for the same trait should be expected in different years because QTL detection will depend on the prevailing yearly environmental variations. This will result in an increased number of QTLs and affect the detection of different QTLs across years for each fruit trait. Out of the seven QTL clusters, five harbored at least one stable QTL.

Robust QTLs or QTL clusters in multiple environments provide valuable information for further underlying gene identification. The QTL clusters are QTL-rich regions containing two or more QTLs of different traits within some common confidence-overlapping region. We found seven QTL clusters with stable or common QTLs affecting two or more different traits distributed on six chromosomes (LG1, LG2, LG3, LG6, LG9, and LG11). Four TSS- and NRS-QTL regions (TNs) containing TSS- and NRS-QTLs were found to affect two or more different traits in the segregants. Cluster-1 (C1), located near mPgCIR8, affected TSS (62.17 cM) and TA (61.52 cM), whereas C2 affected VC, TS, RS, and NRS, located near Pg_PC01_KASP19 and Pg_PC01_KASP26 on LG1. C5 affects TS and RS, located near AS/PL_InDel-30 (47.40 cM) in more than one environment (**Table 5**). These QTL clusters are very important and noteworthy, especially the QTL cluster C2 affecting more than two fruit-quality traits (**Figures 4**, **5**). This genetic map of 'AS' x 'PG' successfully lays the foundation for further fine mapping of these QTLs. QTL analyses of fruit quality in a guava F_1_ population developed from an intraspecific cross demonstrates the extent to which transgressive segregation for fruit biochemical traits can occur in intraspecific progenies derived from plant types with standard phenotypic values. Pyramiding of favorable alleles in the QTLs from the introgression segment of 'AS' and 'PG' has the potential to greatly improve fruit quality traits (i.e., TSS, TA, VC, TS, and RS) and NRS in guava varieties through marker-assisted breeding. The 18 superior segregant individuals obtained in our study lays the foundation for the further fine mapping of traits in subsequent filial generations.

## Conclusion

To the best of our knowledge, this is the first report of a constructed linkage map in guava based on genome-wide SNP, SSR, and InDel markers. This linkage map is a worthy reference for the fine mapping of important fruit traits in guava. Continuous phenotypic variation displayed in the segregating progeny reflects the genetic differences between the phenotypically contrasting parents: ‘Allahabad Safeda’ was superior in TS, TSS, RS, and VC, and Purple Guava was predominant for NRS and polygenic inheritance of these traits. QTLs with major effects on fruit quality, specifically the seven QTL-rich regions affecting two or more different traits and 13 QTLs detected in multiple environments, indicate that genetic background has a stronger effect on fruit-quality traits than environmental factors. In addition, it also demonstrates that genetic and environmental interactions surely effect the fruit quality in guava, as in other species. Hence, the identification of molecular markers associated with fruit-quality traits might prove useful in facilitating future marker-assisted breeding in guava. Therefore, our study provides valuable information and new stable QTL regions for undertaking MAS in guava. Overall, our study has extended knowledge on the inheritance and genetic controls for key guava fruit-quality traits and provided eight superior transgressive segregants that can be evaluated for agronomic characters over different agroclimatic zones.

## Data availability statement

The original contributions presented in the study are included in the article/**Supplementary Material**. Further inquiries can be directed to the corresponding authors.

## Author contributions

SM wrote the manuscript draft. AM and JB edited manuscript draft. JB, AM, MG, NA, and SM designed the experiments, and managed field planting and phenotypic identification. SM, NS, GD, HS, and ST performed the data analysis, and prepared the figures and/or tables. PC, AM, and JB supervised the research and provided the resources. All authors contributed to the article and approved the submitted version.
