# Supplementary material for: Construction of a genetic linkage map and QTL mapping of fruit quality traits in guava (Psidium guajava L.)
Source: Front Plant Sci. 2023 Jun 22;14:1123274. doi: 10.3389/fpls.2023.1123274 (PMC10324979; doi:10.3389/fpls.2023.1123274)
Supplement: Supplementary file 1 [file DataSheet_1.docx]

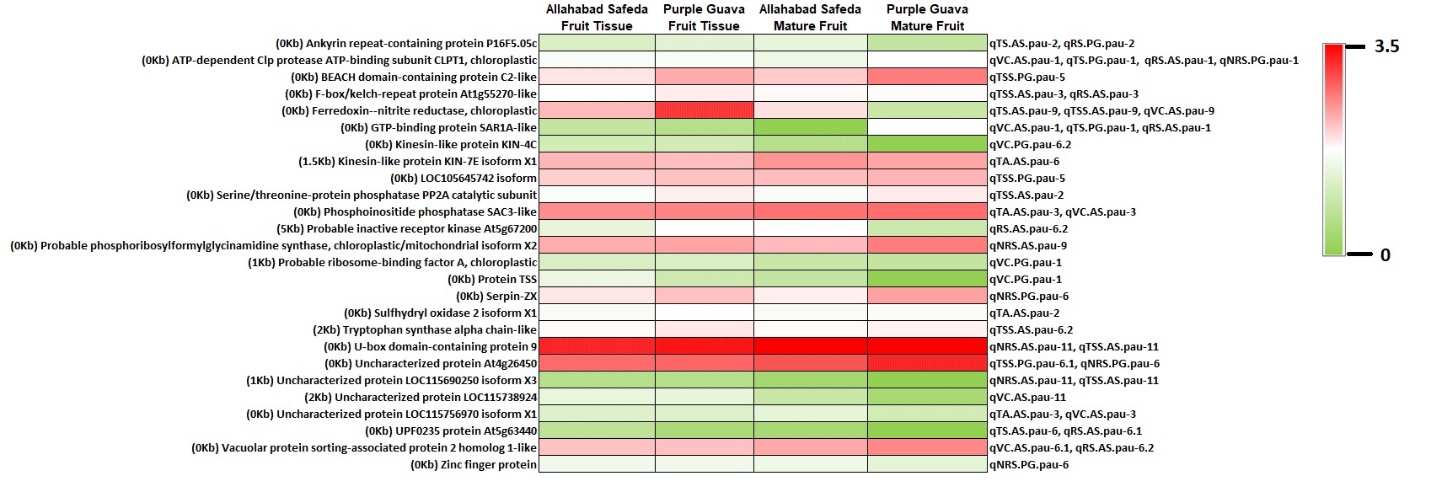


Supplementary Figure 1: Heat map of differentially expressed genes associated to fruit quality related QTLs in Allahabad Safeda and purple guava. The reads were converted from FPKM (Fragments per kilo base of transcript per million mapped fragments) value to the log scale for better visualization.
